# Expression and Characterization of L-Arabinose Isomerase and Its Enzymatic Recycling of the Expired Milk

**DOI:** 10.3390/foods14111873

**Published:** 2025-05-25

**Authors:** Zhou Chen, Yuhan Yan, Ziang Wu, Yanyin Song, Jiangqi Xu

**Affiliations:** School of Food and Health, Beijing Technology and Business University, No.11 Fucheng Road, Beijing 100048, China; zhouch2017@btbu.edu.cn (Z.C.); 15911108313@126.com (Y.Y.); m13146241562_1@163.com (Z.W.); songyanyin0214@163.com (Y.S.)

**Keywords:** L-arabinose isomerase, D-tagatose, expired milk, *Bifidobacterium moukalabense*, rare sugars

## Abstract

As global milk production continues to rise, the disposal of expired milk contributes to environmental pollution and valuable resource wastage. This study presents the development of a novel L-arabinose isomerase, designated *Bm*AIase12, and its application in the enzymatic recycling of expired milk. *Bm*AIase12 exhibited a specific activity of 10.7 U/mg and showed optimal performance at 50 °C and pH 7.0. Furthermore, it exhibited higher activity than most other L-arabinose isomerases. It converted D-galactose into D-tagatose with a high conversion ratio of 53.3% after 48 h at 50 °C. The conversion efficiency of expired milk to D-tagatose was recorded at 40.62%, resulting in a maximum tagatose yield of 1.625 g/L. This was accomplished through the incorporation of β-galactosidase (120 U/mL) and *Saccharomyces cerevisiae* (30 mg/mL) to hydrolyze lactose and metabolize glucose, followed by the addition of 3 U/mL of *Bm*AIase12. Ultimately, following purification, the purity of tagatose was determined to be 98%, and the final yield was 29.8%. These results suggest that *Bm*AIase12 may serve as a promising enzyme for D-tagatose production due to its high conversion yield.

## 1. Introduction

The dairy industry has an important position among the food industries [1]. However, with the gradual increase in dairy production, milk and other processed dairy products have become a major component of much food waste. Annually, over 10 million tons of dairy products are wasted worldwide [2]. Considering their high organic content of milk protein and lactose, the disposal of expired dairy products represents a significant challenge that has considerable implications for both human health and the environment [3]. In light of this issue, research has commenced on methods to recycle spoiled milk, including the development of multifunctional waterproof coatings utilizing proteins derived from expired milk [4]. Additionally, lactose extracted from expired milk can be employed in the production of ethanol fuel [5]. However, the current applications of expired milk in food research remain limited. An increasing number of studies have demonstrated that expired milk and other dairy by-products are considered inexpensive materials and evaluated as alternative substrates in microbial production processes [6]. For example, expired milk can be used to extract nutritional components for cultivating edible fungi. [7]. These biological processes can yield valuable compounds, including nisin, biomass, and lactic acid, which contribute to sustainable consumption and production. The sugars in expired milk are more stable than perishable proteins, making them suitable as the primary resource for recycling lactose and galactose. Unspoiled milk can be processed to produce a large amount of whey powder, which contains 44–50 g/L of lactose [8]. However, these studies have focused on producing low-value products, thereby failing to significantly improve economic viability. There is still great potential for the high-value conversion of lactose from expired milk in the dairy industry.

Recently, there has been an increase in the production of rare sugars from various monosaccharides because of their biological functions suitable for industrial applications, including in the food, cosmetics, and pharmaceutical industries [9]. The International Society for Rare Sugars (ISRS) defines rare sugars as monosaccharides and their derivatives that are rarely found in nature [10]. D-tagatose is a naturally occurring rare sugar originally found in gum extracts and lichens, but also in dairy products [11]. D-tagatose has the same taste as sucrose, with up to 92% sweetness [12] but produces only 1.5 kcal/g (only 30% of the energy content of sucrose) [13], which is far less than the calories provided by sucrose. Therefore, D-tagatose is considered a low-calorie sweetener and an isomer of D-galactose, serving as a potential sucrose replacement [13]. D-tagatose has been demonstrated to be non-cariogenic, as it does not promote plaque formation in contrast to sucrose [14]. Furthermore, D-tagatose exhibits properties that may prevent dental caries and obesity, reduce blood glucose levels, and confer positive effects on gastrointestinal health [15]. The U.S. Food and Drug Administration added D-tagatose to the GRAS substance list, and its use in food has since been recognized by many countries [16]. Given the rising demand for tagatose, the development of high-conversion preparation methods is essential for future advancements. Consequently, there is an urgent need to establish efficient and scalable techniques for the large-scale synthesis of tagatose.

Using a chemical or an enzyme catalyst, D-tagatose can be produced from D-galactose [17]. Due to the several disadvantages inherent in this process, the enzymatic route is preferred, and L-arabinose isomerase (L-AI, EC 5.3.1.4) is the enzyme most frequently used for this purpose [18]. Currently, L-AI has been found and cloned from about 30 distinct bacterial species [19], including genera belonging to *Lactobacillus*, *Bacillus*, *Geobacillus*, *Thermotoga*, and *Thermoanaerobacterium,* among others [20]. The majority of the identified L-AIS exhibits significant potential for the production of D-tagatose at the laboratory scale. However, for industrial applications, the use of enzymes with high conversion efficiency is of paramount importance. In previous studies, the conversion ratio of L-AI to D-galactose to D-tagatose was low, generally around 20–35%. For example, *Lactobacillus rhamnosus* showed a conversion yield of 29.3% [21], *Bacillus stearothermophilus* 33.3% [22], and *Arthrobacter* sp. 22c 30% [23]. Previous studies explored a highly D-galactose-specific L-AI from *Bifidobacterium adolescentis* for the production of D-tagatose [24]. Salonen et al. [25] reported that the use of purified *Bifidobacterium longum* L-AI as a catalyst at a temperature of 35 °C resulted in a ratio of 36% D-tagatose. We speculate that the high transformative capacity of L-AI from other bifidobacterial sources is similar to findings from prior research. Consequently, our objective was to identify an enzyme that effectively facilitates the production of D-tagatose.

In this study, we present the first report on an L-arabinose isomerase (L-AI) derived from *Bifidobacterium moukalabense* (*Bm*AIase12). The enzyme was structurally and biochemically characterized after the cloning and expression of its corresponding gene (araA) in *Escherichia coli* BL21(DE3). The purified enzyme was studied to determine the optimal conditions to obtain its maximum activity and to evaluate its ability to produce D-tagatose. During this process, D-galactose is produced through the hydrolysis of expired milk by the β-galactosidase and *S. cerevisiae* and is then converted into its isomer D-tagatose by *Bm*AIase12. In this research, we successfully accomplished a high-value conversion of lactose derived from expired milk within the dairy sector, thereby enabling the economically viable and efficient synthesis of D-tagatose from dairy waste.

## 2. Materials and Methods

### 2.1. Materials

D-glucose, D-galactose, D-tagatose, L-Cysteine hydrochloride anhydrous, and Carbazole were purchased from Aladdin Biochemical Technology Co. Ltd. (Shanghai, China). Tris, imidazole, kanamycin sulfate, and an unstained Protein Marker were purchased from Biorigin Inc. (Beijing, China). The Lowry kit and Isopropyl β-D-Thiogalactoside (IPTG) were purchased from Beijing Solarbio Science & Technology Co., Ltd. (Beijing, China). The Chelating Sepharose (Ni-IDA) resin matrix was from GE Life Sciences (Pittsburgh, PA, USA). *Saccharomyces cerevisiae* (Instant dry yeast) was acquired from Angel Yeast Co., Ltd., located in Yibin, China. β-Galactosidase (8.0 units/mg solid) from *Aspergillus grace*, was purchased from Azure Sky Biotechnology Co., Ltd. (Beijing, China). Expired milk is a product that has surpassed its designated expiration date of the retail distribution of pasteurized milk in supermarkets from Beijing Sanyuan Food Co., Ltd. (Beijing, China). All other reagents utilized in this study were of analytical or chromatographic grade, unless specified otherwise.

### 2.2. Gene Mining and Cloning of Novel L-Arabinose Isomerase

The BLAST 1.4.0 (Basic Local Alignment Search Tool) analysis was conducted using the NCBI database (https://www.ncbi.nlm.nih.gov/), resulting in the identification of a hypothetical L-arabinose isomerase protein (GenBank WP_137658244.1) sourced from the genomic data of *Bifidobacterium moukalabense*, which has not been characterized through expression. To express the full-length gene of *Bm*AIase12, the gene fragment was further amplified by PCR using two primers containing *NdeI* and *XhoI* sites: *Bm*AIase12 forward: (ATTCTACATATGTACCGTTACCTTTTGGGCAAG) and *Bm*AIase12 reverse: (ATTCCGCTCGAGTCAGTGACGGTTGTTGAGACGGTG). Based on an analysis of restriction enzyme sites in both the target gene and expression plasmid’s polycloning region, *Bm*AIase12 was engineered through in silico design by introducing compatible restriction sites into the target gene’s flanking sequences. Transform the recombinant plasmid containing the gene of interest into competent *E. coli* BL21 (DE3) cells.

### 2.3. Expression and Purification of the BmAIase12

*Bm*AIase12 was inoculated into 10 mL of LB medium (containing 5 g/L yeast extract, 10 g/L tryptone, and 10 g/L NaCl) in a 50 mL flask at 37 °C with shaking at 200 rpm. The fermentation medium was supplemented with kanamycin at a final concentration of 50 mg/mL to maintain the plasmid [26]. The growth of *Bm*AIase12 continued until the optical density (OD_600_) at 600 nm reached 0.6; then, β-D-1-thiogalactopyranoside (IPTG) was introduced to a final concentration of 1 mM for expression, and growth was continued at 25 °C for 24 h [27]. The fermentation cells were collected by centrifugation at 4 °C and 10,000× *g* for 20 min, which were then resuspended in 0.05 mM Tris-HCl buffer (pH 7.4). The bacterial suspension was placed in a 4 °C ice bath and underwent ultrasonic disruption for 10 min at 15% power (maximum power: 600 W), alternating between 2 s of operation and 2 s of pause. After that, the mixture was centrifuged at 10,000× *g* for 20 min at 4 °C to eliminate cell debris, resulting in the extraction of soluble *Bm*AIase12 (crude enzyme) from the supernatant.

For purification of *Bm*AIase12, the supernatant was applied to a Ni-IDA affinity column (0.8 × 10 cm) packed with Ni^2+^-charged Sepharose resin [28]. The column was previously equilibrated with a binding buffer (50 mM Tris-HCl buffer, pH 7.4). Washing buffer (50 mM Tris-HCl buffer, 100 mM imidazole, pH 7.4) was used to remove the nonspecific binding proteins [19]. Elution buffer (50 mM Tris-HCl, 300 mM imidazole, and pH 7.4) was used to obtain the recombinant *Bm*AIase12. The purified enzyme was desalted via ultrafiltration using 10 kDa molecular weight cutoff dialysis membranes (EMD Millipore, Billerica, MA, USA) against 50 mM Na_2_HCO_3_-citric acid buffer (50 mM, pH 7.0), followed by storage at 4 °C. Protein purity and molecular weight were analyzed by 10% SDS-PAGE.

### 2.4. Enzyme Assay and Protein Determination

The catalytic activity of *Bm*AIase12 was determined by measuring the cysteine–sulfuric acid–carbazole method. The reaction mixture consisted of 20 μL 0.2M D-galactose, 10 μL purified enzyme, and 70 μL of 50 mM Na_2_HCO_3_-citric acid buffer at a pH of 7.0, unless otherwise specified. The mixtures were incubated statically at a temperature of 50 °C for 10 min. After that, the reactions were halted by subjecting the mixtures to boiling water for 10 min. The concentration of D-tagatose was measured using the cysteine–sulfuric acid–coumarin method through ultraviolet spectrophotometry. The experiment was repeated three times in parallel [29]. One unit of enzyme activity (U) was defined as the amount of enzyme required to produce 1 μmol of D-tagatose per minute under the specified reaction conditions.

The protein concentration was determined by the Lowry assay [30], employing bovine serum albumin (BSA) as the standard protein reference. The absorbance measurements were performed at 650 nm using a spectrophotometer (Agilent Technologies, Santa Clara, CA, USA).

### 2.5. Temperature and pH Profiles

The optimal reaction temperature of *Bm*AIase12 was determined by varying the reaction temperature (30–65 °C). To evaluate its thermal stability, the enzyme solution was incubated in different temperature (30–65 °C) for 30 min. Subsequently, the solution was rapidly cooled to 0 °C in order to evaluate the residual activity through a standardized assay.

To characterize the properties of the purified enzyme across varying pH conditions, the enzyme solution was assayed at pH values ranging from 3.0 to 9.0 using 100 mM buffer systems composed of citrate–phosphate and Tris-HCl buffers at the optimal temperature [28]. Enzyme activity was measured in these pH reaction systems to determine the optimum pH. In order to evaluate the pH stability of the enzyme solution, it was diluted with various pH buffers, specifically citrate–phosphate buffers ranging from pH 3.0 to 7.0 and Tris-HCl buffers from pH 7.5 to 9.0. The diluted enzyme solutions were subsequently incubated in a water bath maintained at 50 °C for 30 min. Subsequently, the solution was rapidly cooled to 0 °C in order to evaluate the residual activity through a standardized assay.

### 2.6. Influence of Metal Ions

The effects of different modulators, such as metal ions and chelating agents, on the activity of *Bm*AIase12 were investigated. The enzyme was incubated with 1 mM of Ca^2+^, Mg^2+^, Fe^2+^, Fe^3+^, Cu^2+^, Mn^2+^, Zn^2+^, Ni^2+^, Ba^2+^, and Co^2+^ for 30 min, and then the residual enzymatic activity was assessed utilizing a standard methodology. The enzyme’s residual activity was assessed in comparison to the control (without metal ions) through the standard assay method.

### 2.7. Examination of the Ability of BmAIase12 to Catalyze Synthesis of D-Tagatose

The D-tagatose conversion activity of *Bm*AIase12 was characterized by thin-layer chromatography (TLC), with the reaction supernatant being applied to silica gel TLC plates (Merck). N-butanol, ethanol, and water (5:3:2, *v*:*v*:*v*) were combined to create the TLC spreading agent. As a color developer, sulfuric acid and methanol (5:95, *v*:*v*) by mass were added, and 10 mg/mL of D-galactose and D-tagatose standards served as controls.

The chromatographic plate was placed in a developing chamber within a fume hood, and the mobile phase was allowed to migrate until it reached approximately 2 cm from the top of the silica gel plate. The plate was then removed and air-dried naturally.

The pure enzyme solution was prepared as described above. A 1 mL mixed reaction system was set up, consisting of 100 μL of 1 mM galactose, 200 μL of 1 U purified enzyme, and 700 μL of 50 mM Na_2_HCO_3_-citric acid buffer (pH 7). The reaction was carried out at 50 °C (unless otherwise indicated). The yield of tagatose was measured by TLC and the cysteine–sulfuric acid–carbazole method after different reaction times, and the reaction time course curve was plotted.

### 2.8. Enzymatic Recycling of Expired Milk by BmAIase12

The expired milk was centrifuged directly at 10,000× *g* for 10 min, and the supernatant was collected. Subsequently, the transformation products were analyzed using thin-layer chromatography after the addition of β-galactosidase (120 U/mL) and *S. cerevisiae* (30 mg/mL), which were allowed to react overnight at 30 °C. The reaction mixture was boiled in water for 10 min to terminate the reaction, followed by centrifugation at 10,000× *g* for 10 min to remove residual yeast and β-galactosidase precipitates. The pH was adjusted to the optimal value for the enzyme, followed by the addition of purified L-arabinose isomerase to initiate the reaction. The enzyme addition amount was then varied for 2 h at 50 °C and pH 7, while the substrate concentration remained constant. Then, under the condition of optimal enzyme addition, the effect of time on the conversion of galactose to D-tagatose by L-arabinose isomerase was investigated. After that, samples were taken at 1 h, 2 h, 4 h, 8 h, 12 h, 16 h, and 24 h, respectively, and the concentration of D-tagatose in the reaction solutions corresponding to these varying reaction times was quantified. The reaction time course was plotted according to the amount of D-tagatose produced.

### 2.9. Isolation and Purification of D-Tagatose

Following the β-galactosidase and *S. cerevisiae* reaction, *Bm*AIase12 was gradually added to the remaining expired milk, and the transformation products containing D-galactose and D-tagatose were centrifuged by 10,000× *g* for 5 min. In this study, the ion-exchange column was selected as a chromatography column with a length of 1 m and an internal diameter of 1.6 cm. The supernatant was passed through a chromatography column, which was filled with the processed Ca^2+^ chromatography separation resin. A mixture of D-galactose and D-tagatose was eluted using deionized water as the mobile phase, and a partial collector was used to collect the eluted samples.

After collection, the samples were first concentrated by rotary steaming. The concentration of D-tagatose in the concentrated sample was qualitatively and quantitatively detected by thin-layer chromatography (TLC) and high-performance liquid chromatography (HPLC) when compared with the D-tagatose standard sample. The detection conditions of HPLC were as follows: Column: YMC-Pack NH_2_ (4.6 × 250 mm); Column temperature: 30 °C; Mobile phase: 70% acetonitrile and 30% water; Flow rate: 0.6 mL/min; Injection volume: 10 μL; Detector: Refractive index detector.

## 3. Results

### 3.1. BmAIase12 Recombinant Production and Purification

In order to find an effective L-arabinose isomerase for the biosynthesis of D-tagatose, a novel L-arabinose isomerase was designed based on previously reported biosynthetic gene (clusters). In this process, we screened a selection of putative L-arabinose isomerases from *Bifidobacterium moukalabense* within the NCBI database. The *L-AIs* gene from *B. moukalabense* was cloned and subsequently expressed in *E. coli* BL21 (DE3).

**Figure 1:**
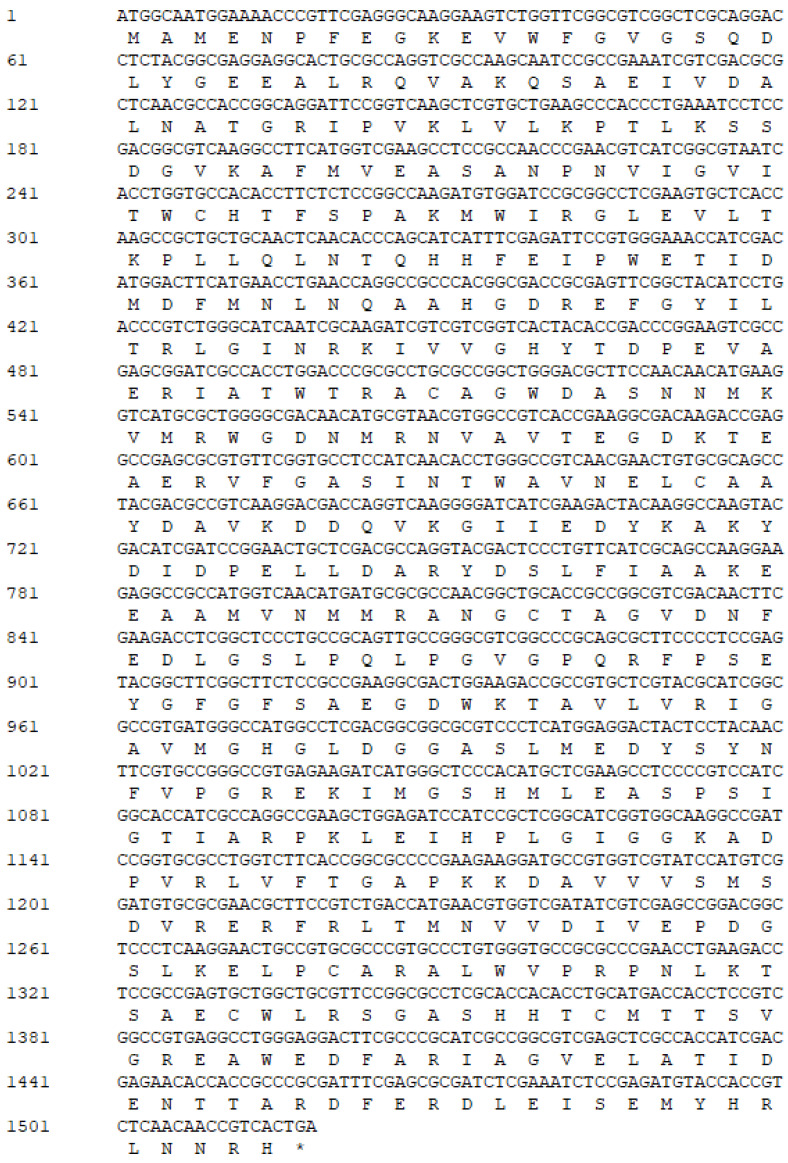
Nucleotide and deduced amino acid sequences of the full-length cDNAs and flanking regions of *Bm*AIase12. The asterisk (*) denotes the stop codon.

In the present study, a L-arabinose isomerase (*L-AIs*) derived from *Bifidobacterium moukalabense* was identified through screening of the NCBI database (https://www.ncbi.nlm.nih.gov/). Nucleotide and deduced amino acid sequences of the full-length cDNAs and flanking regions of *Bm*AIase12 are shown in Figure 1. The complete *Bm*AIase12 gene encompasses an open reading frame of 1515 base pairs, which encodes a protein consisting of 505 amino acid residues, with an anticipated molecular weight of 57.0 kDa.

**Figure 2:**
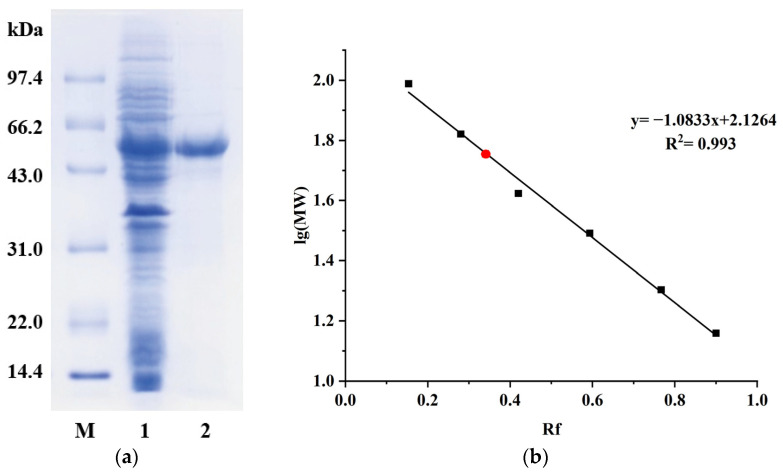
SDS-PAGE analysis of purified *Bm*AIase12 (**a**): Lane M, low molecular weight standard protein markers; lane 1, crude L-arabinose isomerase; lane 2, after Ni-IDA. Standard curve of protein (**b**): Relative mobility of standard proteins (Rf); logarithm of molecular weight of standard proteins (log MW). The red bullet denotes the *Bm*AIase12 enzyme protein.

The *Bm*AIase12 was purified by Ni-IDA affinity chromatography, and the results are shown in Table 1. *Bm*AIase12 was purified to 2.1-fold with a total yield of 59.6% and an increase in specific activity from 5.1 U/mg to 10.7 U/mg. Following SDS-PAGE analysis (Figure 2), the purified L-AI exhibited a single homogeneous protein band with an estimated molecular mass of 57.0 kDa.

### 3.2. Biochemical Characterization of BmAIase12

**Figure 3:**
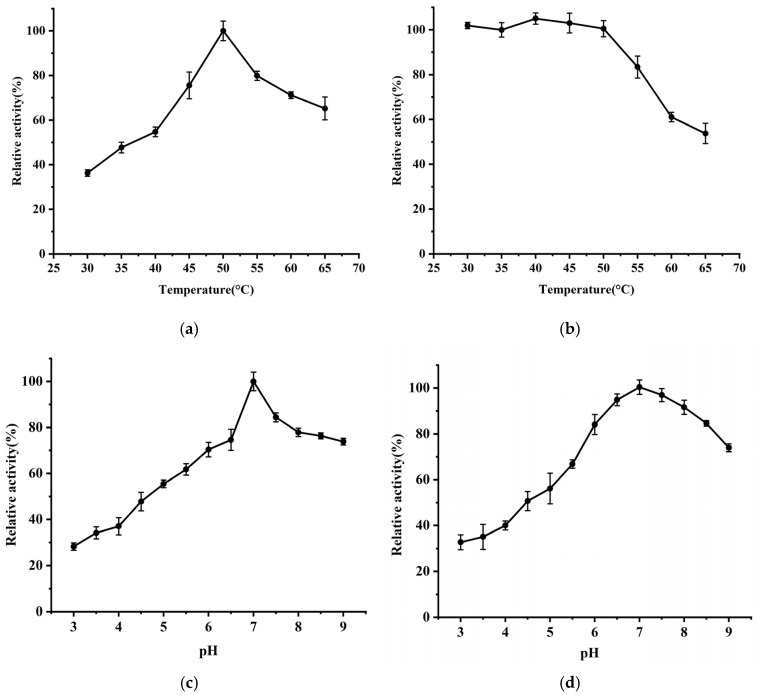
The temperature optima (**a**), the temperature stability (**b**), the pH optima (**c**), and the pH stability (**d**) on the L-arabinose isomerase (L-AI) activity. Experiments for each test were conducted in triplicate and reproducible results were obtained.

The effect of temperature on *Bm*AIase12 activity was investigated by conducting the reactions at different temperatures. As shown in Figure 3a, the enzyme displayed an optimal temperature of 50 °C. And the enzyme was stable up from 30 °C to 65 °C when incubated for 30 min, retaining more than 80% of its initial activity at 55 °C (Figure 3b). The enzymatic activity and stability of *Bm*AIase12 were further investigated at a pH range of 4.0–9.0 (Figure 3c). *Bm*AIase12 exhibited maximal activity at pH 7.0. The enzyme exhibited excellent stability, it retained over 80% of its relative activity between pH 6.0 and 8.5 (Figure 3d).

### 3.3. Influence of Metal Ions

**Figure 4:**
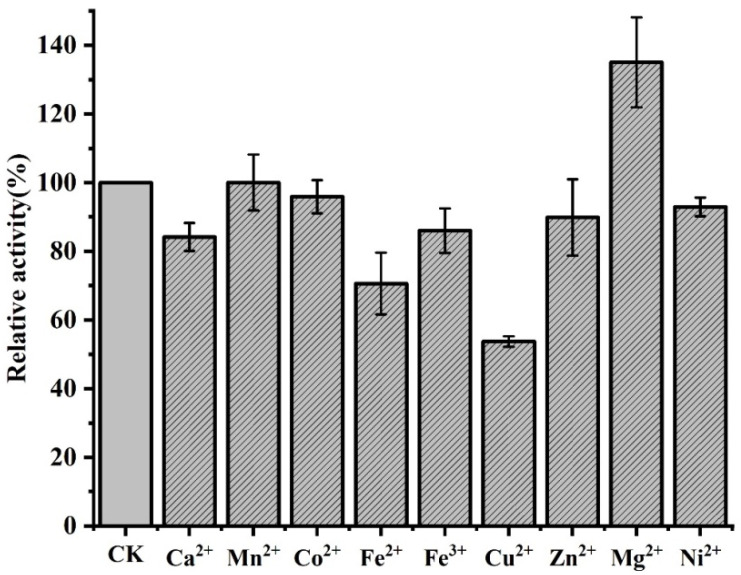
Effects of metal ions on the L-arabinose isomerase (L-AI) activity. Experiments for each test were conducted in triplicate and reproducible results were obtained. CK is regarded as a control check (Blank control).

Several divalent metal ions were added at a final concentration of 1 mM in order to examine the relative activity of *Bm*AIase12 in their presence. According to Figure 4, Fe^2+^(70%), Fe^3+^(86%), and Zn^2+^(89%) marginally decreased the enzymatic activity of *Bm*AIase12, whereas low doses of Cu^2+^ (53%) greatly hindered the activity. In contrast, the enzymatic activity of *Bm*AIase12 may be activated by Mg^2+^ (135%), Ba^2+^ (102%), and Mn^2+^ (101%). The activity was not significantly impacted by other chemical substances, including Co^2+^, Ca^2+^, and Ni^2+^.

### 3.4. Enzymatic Synthesis of D-Tagatose by BmAIase12

**Figure 5:**
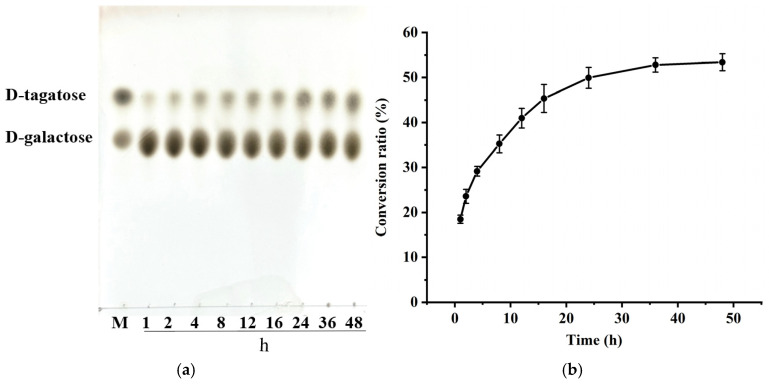
TLC (**a**) and HPLC (**b**) determination of D-tagatose production. Lane M, a mixture of D-tagatose and D-galactose.

In this study, *Bm*AIase12 was efficiently converted into D-galactose to produce D-tagatose. The enzymatic reactions were performed with varying incubation times, as illustrated in Figure 5a. D-tagatose generation progressively rises as reaction time increases. As shown in Figure 5b, purified *Bm*AIase12 catalyzed 100 mmol/L D-galactose with a maximum conversion ratio of 53.3% after 48 h at 50 °C and pH 7.0.

### 3.5. Capacity of BmAIase12 to Enhance the Conversion of Expired Milk

Over time, whey permeates, and protein precipitates can be formed from expired milk. The concentration of lactose in the whey permeate, measured by HPLC after centrifugation, is about 20 g/L. After adding 120 U/mL of β-galactosidase and 30 g/mL of *S. cerevisiae* to the whey permeate, the mixture completely reacted at 40 °C for 12 h, hydrolyzing all of the lactose and consuming the glucose.

**Figure 6:**
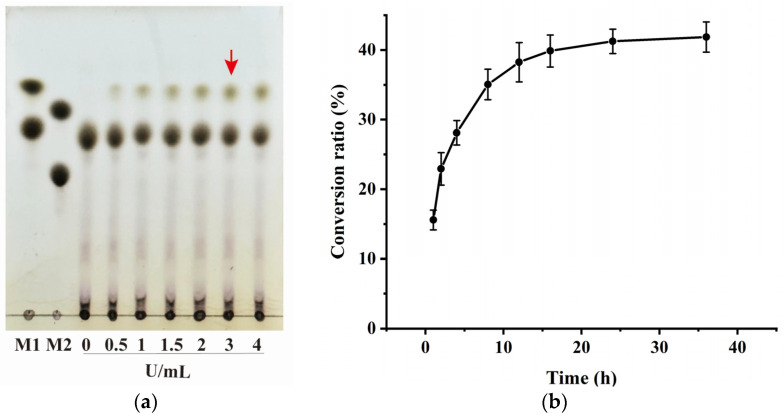
TLC (**a**) determination of the conversion of expired milk to the effect of enzyme addition on the amount of D-tagatose produced. The red arrows indicate the optimal enzyme dosage for tagatose production. The conversion ratio of D-tagatose from D-galactose by *Bm*AIase12 in the expired milk (**b**).

The effect of different *Bm*AIase12 doses (0.5–4 U/mL) on galactose-converted tagatose produced from expired milk was investigated (Figure 6a). As 0~3 U/mL *Bm*AIase12 was added, the D-tagatose concentration grew progressively before tending to stabilize. Further increases in the amount of enzyme given have little influence on the rate of reaction once they are beyond the critical value. Since no significant increase in D-tagatose production was observed with the addition of 4 U/mL *Bm*AIase12, 3 U/mL was selected as the optimal enzyme loading for subsequent experiments. This is also consistent with the article’s claim that high L-AI concentrations can increase yields and reduce enzyme activity failure [22].

The generation of tagatose was determined at different reaction times (1–36 h). With the extension of the reaction time, the yield of tagatose increased significantly. The pace at which D-tagatose was produced was rapid from 1 to 12 h. After 12 h, the rate of reaction slowed and then steadied, and after 36 h (Figure 6b), *Bm*AIase12 catalyzed the conversion of galactose produced from 40.62% expired milk into D-tagatose, achieving a maximum D-tagatose yield of 1.625 g/L.

### 3.6. Isolation and Purification of the Converted D-Tagatose

**Figure 7:**
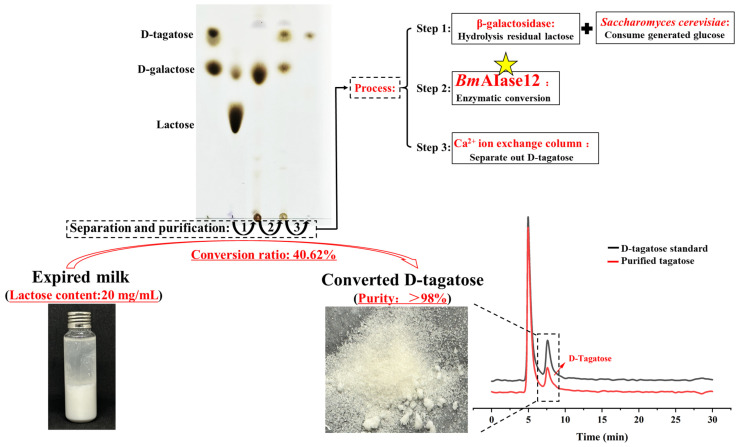
Flowchart of the purification process of converted D-tagatose from the expired milk.

The tagatose derived from the previously mentioned expired milk was isolated and purified using calcium ion-exchange chromatography. The samples containing D-tagatose and D-galactose were separated by the column, which was filled with a processed Ca^2+^ ion-exchange resin column. Figure 7 displays the separation process. Through HPLC analysis, it was shown that the concentration of D-tagatose was more than 98%. Using expired milk as substrate, the concentration of 3.58 mg/mL tagatose was obtained after the above steps, plus enzymatic reaction and purification, and the final yield of D-tagatose was 29.8%.

## 4. Discussion

Lactic acid bacteria are widely employed in the food industry [25], particularly in the production of fermented foods. L-AIs isolated from lactic acid bacteria generally demonstrate a low functional pH range [31]. However, there are not many L-AIs that have been fully characterized by lactic acid bacteria. Notably, *Bifidobacteria* are among the most significant physiological bacteria present in the intestinal tracts of both humans and animals, fulfilling numerous essential physiological functions. Among them, *Bifidobacterium moukalabense* is a novel species of genus *Bifidobacterium* isolated from the feces of a wild lowland gorilla [32]. Herein, a BLAST homology search for *Bm*AIase12 amino acids showed good novelty, but this cloned gene has not been further studied. SDS–PAGE analysis of the extracts of *E. coli* BL21 cells, revealed the presence of protein with a molecular weight of 57 kDa, which was similar to the molecular weights of other reported L-AIs, such as 56 kDa in *L. rhamnosu*s [21], 55 kDa in *B. longum* [25], and 57 kDa in *T. neapolitana* [33]. The activity of the purified *Bm*AIase12 was found to be 5.4 U/mg. Previous studies have reported different activity of L-AIs depending on the source strains. The activity of most L-AIs reported in the literature is between 0.42 and 24.47 U/mg [34,35,36]. The purified *Bm*AIase12 has a higher specific enzyme activity, which may promote the conversion of D-galactose.

Thermodynamic equilibrium limits isomerase-catalyzed processes. While elevating the reaction temperature may favor the formation of products by shifting the reaction equilibrium, excessively high temperatures can diminish enzyme activity and promote the creation of browning sugars [37], thereby compromising product quality, particularly in alkaline environments [38]. The optimum temperature for the highest activity of *Bm*AIase12 was determined to be 50 °C. The optimum temperature for L-AI of BAAI (55 °C) [24] was also reported to be similar. *L. plantarum* [39] and *Lactobacillus fermentum* [40], two other lactic acid bacteria, exhibited similar temperature optimums of 50–65 °C. After 30 min of incubation at 45–55 °C, *Bm*AIase12 still exhibited over 80% residual enzyme activity. After 30 min of incubation at 60 °C, the residual enzyme activity started to decline, although 50% of the enzyme activity was still present at 65 °C. According to the results, the L-AI stability suffers at high temperatures, consistent with other literature reports [25].

The activity of L-AI at a weakly acidic pH is a characteristic required for the production of D-tagatose [41]. The optimum pH value for the majority of previously identified L-AIs is between 7.0 and 8.5, such as *Bacillus amyloliquefaciens* (pH 7.5) [42], *Bacillus thermoglucosidasius* (pH 7.0) [43], and *Enterococcus faecium* (pH 7.0) [18]. Examination of the relationship between pH and *Bm*AIase12 activity revealed that the enzyme was most active at pH 7.0; it only displayed 70% of its maximum activity at pH 6.0. After 30 min of incubation at various pH, the remaining enzyme activities were measured to determine the pH stability, and the *Bm*AIase12 was shown to be highly stable between pH 6.0 and 8.0. The stability of purified *Bm*AIase12 in the weakly acidic pH range is important for industrial applications.

Overall, most studies conclude that Mn^2+^ and Co^2+^ are co-cofactors for various L-AIs activities [43]. However, Mn^2+^ and Co^2+^ did not significantly enhance the catalytic activity of *Bm*AIase12 in this study, which differed from the reported results [44,45]. Cu^2+^ exhibits the highest inhibition in many previous studies, which is consistent with our findings [20]. Most reported L-AIs tend to increase their activity with Co^2+^ as a cofactor [13]. Cobalt is a heavy metal that can cause toxicity and disease in humans. In particular, *Bm*Ara12 has a unique metallic preference for Mg^2+^ ions, which has a huge advantage in the industrial production of D-tagatose.

In previous studies, the conversion ratio of L-AI to D-galactose to D-tagatose was low, typically around 20–35%, such as in *Bacillus stearothermophilus* (33.3% [22]) and *Arthrobacter* sp. 22c (30% [23]). The L-arabinose isomerase from *Lactobacillus rhamnosus* can convert D-tagatose with a maximum conversion ratio of 29.30% [21]. Salonen et al. [25] using purified *Bifidobacterium longum* L-AI as the catalyst at 35 °C, yielded 36% D-tagatose. The L-arabinose isomerase from *Lactobacillus* conversion ratio of D-tagatose from 300 g/L lactose achieved 42.4% [28]. Furthermore, research on the high conversion ratio of L-AI catalysis to produce tagatose has been conducted recently. Zhang et al. [24] explored a highly D-galactose-specific L-AI from *Bifidobacterium adolescentis* for the production of D-tagatose. Enzymatic conversion of D-galactose into D-tagatose by L-AI showed 56.7% conversion efficiency. The conversion ratio of this *Bm*AIase12 enzyme for the synthesis of tagatose (53.3%) is better than that of most other studies and holds significant industrial utilization value. This also proves that *Bifidobacterium* is a good source of L-AI, as reported in other studies [24].

Milk has a high nutrient content, and expired milk easily provides a favorable environment for the growth of spoilage microorganisms. Numerous studies indicate that milk, byproducts from milk processing, and expired milk can be repurposed by using various microbial strains [6]. This approach has proven to be a viable method for generating a diverse range of valuable products. Lactose and galactose can be primarily recycled from the sugars in expired milk, which also hold significant potential for other sugar-based preparations. The availability of expired milk as a resource is constrained by the requirements of dairy marketing. Lactose and galactose are more appropriate raw materials for the enzymatic production of purified, value-added food products [46]. In previous studies, a three-stage process had been employed to convert lactose and galactose present in cheese whey permeates into tagatose [22]. Kim et al. [47] produced D-tagatose from the onion juice residue (OJR), which is a major resource for producing monosaccharides such as D-galactose and D-glucose. However, no studies have reported using expired milk as a substrate for the direct enzymatic conversion of its sugars into rare sugars, particularly D-tagatose. The value-added transformation of expired milk is achieved by the *Bm*AIase12 investigated in this work, which is the first report of its kind in the literature. This study employed expired milk as the substrate source, achieving high-value utilization of waste lactose. Compared to the conventional D-tagatose production method using D-galactose as the raw material, this approach demonstrated significantly improved economic feasibility. Notably, the preprocessing steps for expired milk were relatively complex, and batch-to-batch variations in milk composition were observed to potentially impact conversion efficiency. Overall, there was a significant rate of *Bm*AIase12 conversion from expired milk to D-tagatose. In this context, our system may be viable for production on an industrial scale.

A previous study recycled the preparation of high-purity tagatose from galactose using one-pot boronate affinity adsorbent-based adsorption-assisted isomerization and simultaneous purification. The tagatose purity reached ~ 85% with a promising recovery ratio of 87.42% [48]. Hong et al. reported a method to obtain high-purity D-tagatose by ion-exchange chromatography [34]. Huang et al. reported the separation of D-galactose and D-tagatose using Ca^2+^ ion-exchange resins, achieving 98% D-tagatose purity and an 83% recovery rate. Xu et al. used an Amberite column with a water solvent system to separate D-tagatose, thereby avoiding environmental drawbacks. [39]. Considering that the downstream treatment is time-consuming and energy-intensive, and the chemical catalysts used are difficult to separate or recycle, these factors ultimately hinder its sustainable development. In this experiment, a straightforward and environmentally sustainable column chromatography separation method was selected.

The D-tagatose sample separated and desalted by Ca^2+^ ion-exchange column chromatography was enriched and evaporated at 50 °C to concentrate the collected solution. The concentrated D-tagatose was frozen in a refrigerator until solidified, preparing it for subsequent freeze-drying. A part of the freeze-dried sample was dissolved in water for HPLC analysis, and the HPLC spectrum was obtained, as shown in Figure 7. According to the analysis, the D-tagatose sample obtained by enzymatic conversion in this experiment exhibits extremely high purity. This study demonstrates the transformation and purification of high-purity D-tagatose from expired milk, which meets commercial purchase standards and proposes a new approach for the value-added conversion of expired dairy waste. Additionally, the results confirm the feasibility of the separation and purification method, which showed excellent separation efficiency, and suggested its potential for scaling up in industrial D-tagatose production.

## 5. Conclusions

In summary, the L-AI gene from the strain *B. moukalabense* was successfully cloned and expressed as a recombinant protein in *E. coli*. Compared to other L-arabinose isomerases (L-AIs), *Bm*AIase12 not only demonstrates superior thermal stability at medium and high temperatures but also exhibits relatively high activity and stability at acidic pH levels. The successful identification and overexpression of *Bm*AIase12 have enabled the characterization of a novel L-AI with high specificity for galactose. Furthermore, its capacity to convert D-galactose to D-tagatose from expired milk waste indicates significant potential for industrial applications. This study presents a method that significantly reduces raw material costs and enhances waste value. The method lays the foundation for establishing an economically viable and environmentally sustainable sweetener production system while proposing new strategies to address global food waste challenges.

## Figures and Tables

**Table 1:** Purification Summary of the *Bm*AIase12.

Purification Step	Total Activity	Protein	Specific Activity	Purification	Recovery
	(U) ^a^	(mg) ^b^	(Units/mg)	Factor (-Fold)	(%)
crude supernatant	452.5	90.0	5.1	1.0	100.0
Ni-IDA affinity chromatography	269.8	25.0	10.7	2.1	59.6

^a^ Activity was measured in Na_2_HCO_3_-citric acid buffer (50 mM, pH 7) at 50 °C. ^b^ The protein was measured by the Lowry method, using BSA as the standard. Purification factor = Specific activity (Ni-IDA)/Specific activity (crude supernatant). Recovery = Total activity (Ni-IDA)/Total activity (crude supernatant).

## Data Availability

The original contributions presented in this study are included in the article. Further inquiries can be directed to the corresponding author.
